# Adnexal Masses Treated Using a Combination of the SILS Port and Noncurved Straight Laparoscopic Instruments: Turkish Experience and Review of the Literature

**DOI:** 10.1155/2013/836380

**Published:** 2013-11-11

**Authors:** Polat Dursun, Tugan Tezcaner, Hulusi B. Zeyneloglu, Irem Alyazıcı, Ali Haberal, Ali Ayhan

**Affiliations:** ^1^Department of Obstetrics and Gynecology, Baskent University, School of Medicine, Ankara, Turkey; ^2^Department of Surgery, Baskent University, School of Medicine, Ankara, Turkey

## Abstract

*Objective*. To report our experience treating adnexal masses using a combination of the SILS port and straight nonroticulating laparoscopic instruments. *Study Design*. This prospective feasibility study included 14 women with symptomatic and persistent adnexal masses. Removal of adnexal masses via single-incision laparoscopic surgery using a combination of the SILS port and straight nonroticulating laparoscopic instruments was performed. *Results*. All of the patients had symptomatic complex adnexal masses. Mean age of the patients was 38.4 years (range: 21–61 years) and mean duration of surgery was 71 min (range: 45–130 min). All surgeries were performed using nonroticulating straight laparoscopic instruments. Mean tumor diameter was 6 cm (range: 5–12 cm). All patient pathology reports were benign. None of the patients converted to laparotomy. All the patients were discharged on postoperative d1. Postoperatively, all the patients were satisfied with their incision and cosmetic results. *Conclusion*. All 14 patients were successfully treated using standard, straight nonroticulating laparoscopic instruments via the SILS port. This procedure can reduce the cost of treatment, which may eventually lead to more widespread use of the SILS port approach. Furthermore, concomitant surgical procedures are possible using this approach. However, properly designed comparative studies with single port and classic laparoscopic surgery are urgently needed.

## 1. Introduction

Adnexal masses are one of the most common indications for surgery in gynecology clinics, and laparoscopy is generally accepted as the gold standard treatment. Classical laparoscopic surgery for adnexal masses is generally performed using ≥3 trocars. On the other hand, single-port access surgery (SPAS), also known as laparoendoscopic single-site surgery (LESS) and single-incision laparoscopic surgery (SILS), is an evolving endoscopic approach for minimal access surgery. Various surgical procedures, including appendectomy, cholecystectomy, nephrectomy, oophorectomy, hysterectomy, adrenalectomy, gastric bypass, Nissen fundoplication, hernia repair, splenectomy, and colon resection, have been performed via SILS. SILS can result in better cosmesis, shorter recovery time, and less pain than conventional laparoscopy, which requires use of multiple trocar incisions [1, 2].

It was recently reported that adnexal masses could also be treated via SILS [3, 4]. Endoscopic surgery conducted via 3 special luminal ports, including the SILS port (Covidien, Norwalk, CT), GelPort (Applied Medical Resources, Rancho Santa Margarita, CA), and X-cone (Karl Storz, Tuttlingen, Germany), as well as others, is frequently referred to as SILS. SILS requires a 2-3 cm incision on the umbilicus for the placement of the special port. Furthermore, nonconventional roticulating and articulated laparoscopic instruments are necessary for SILS in order to ensure that the instruments do not collide during SILS [5, 6].

SILS performed using conventional laparoscopic instruments for appendectomy and cholecystectomy has been reported; however, to the best of our knowledge, the combined use of the SILS port (Covidien, Norwalk, CT) and conventional laparoscopic instruments has not been reported in the gynecology literature [6, 7]. Herein we report on 14 patients with adnexal masses that were treated using the SILS port and conventional straight laparoscopic instruments.

## 2. Materials and Methods

### 2.1. Participants

The study included 14 women with symptomatic and persistent adnexal masses. Inclusion criteria were as follows: a persistent adnexal mass, a growing adnexal mass on follow-up, an adnexal mass that cannot exclude surgical emergencies, cystic rupture with acute abdomen, and an adnexal mass with intractable pelvic pain. Patients with imaging studies strongly suggesting a malignant adnexal mass were excluded from the study.

### 2.2. Surgical Technique

**Figure 1:**
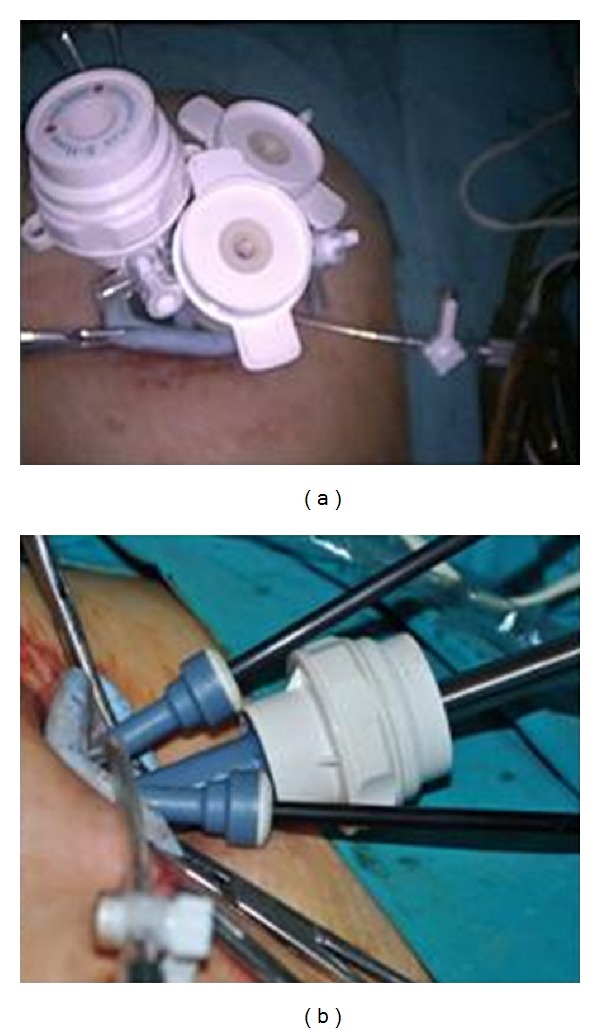
SILS port and instruments positions.

Each patient was placed in the modified lithotomy position under general anesthesia. Initially, the surgeon stood on the left side of each patient. The lateral sides of the umbilicus were everted using 2 clamps. Then, a 2 cm vertical intraumbilical skin incision was made (Figure 1). Sharp and blunt dissection was performed on the subcutaneous fatty tissue; the fascia was exposed and cut using number 11 scalpel blade, and the peritoneum was incised using Metzenbaum scissors. The incision was then extended by an additional 0.5 cm via stretching of the skin. No other extraumbilical skin incisions were used.

**Figure 2:**
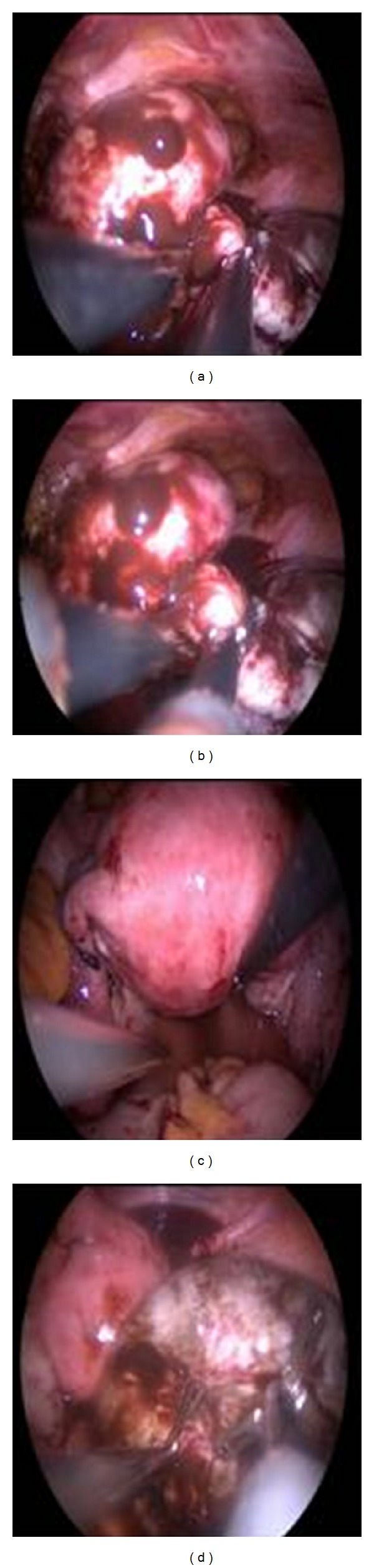
Intraoperative positions of different straight nonroticulating instruments during operations.

**Figure 3:**
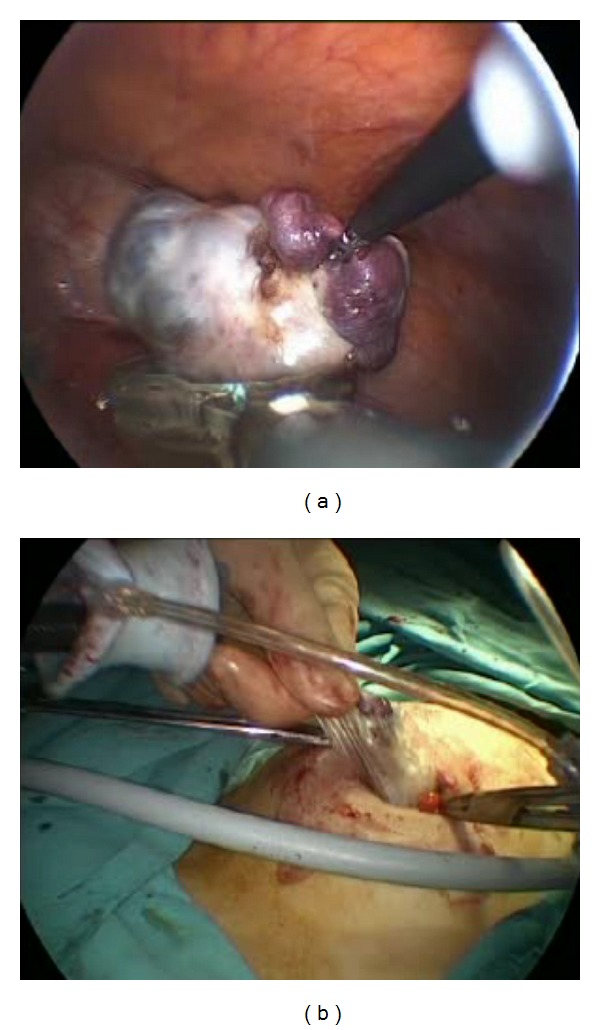
(a) USO material inserted into endobag. (b) Specimen extraction using endobag.

A SILS port (Covidien, Norwalk, CT) with 3 access inlets was inserted into the abdominal cavity using a Heaney clamp, and a carbon dioxide pneumoperitoneum was created. A 10 mm rigid video laparoscope was used together with 2 classical nonroticulating straight laparoscopic instruments (Figure 1). One bipolar and 1 monopolar cautery, 1 dissection forceps, and suction-irrigation devices were used sequentially as indicated during surgery. If collision of the instruments resulted in inadequate surgical movement for dissection, cutting, or coagulation, the surgeon changed the placement of the instruments, his position from the lateral side of the patient to the patient's head, or the placement of the endoscope in order to perform the necessary movements (Figure 2). Specimens were retracted from the umbilical incision at the end of each surgery. If there was a suspicious mass for malignancy, specimen was retracted using endobag via umbilical incision (Figure 3).

The fascia was then closed using number 1 vicryl interrupted sutures. After surgery all patients reported that they are very satisfied with their incision. All surgical procedures were performed by 1 surgeon (PD), except for appendectomy and cholecystectomy, which were performed by a general surgeon (TT).

## 3. Results

**Figure 4:**
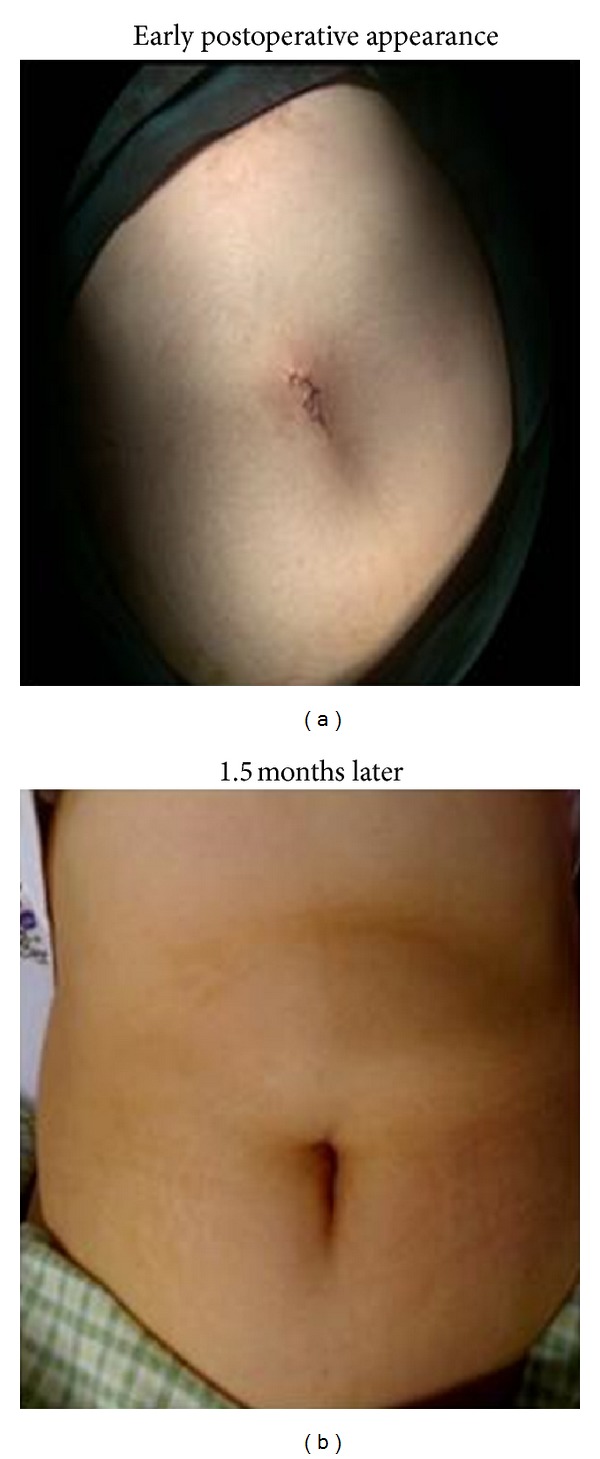
Final appearance at the end of the operation and 1–5 months later.

**Figure 5:**
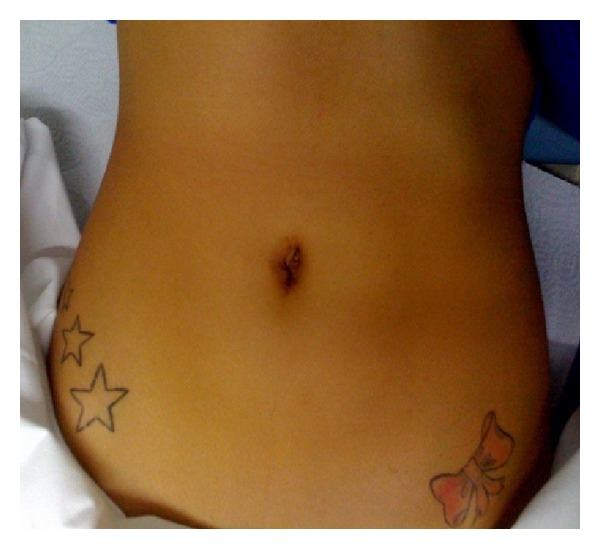
Scar of SILS cystectomy, appearance at 6 months.

Patient characteristics are shown in Table 1. Briefly, all 14 patients had symptomatic complex adnexal masses. Mean age of the patients was 38.4 years and mean duration of surgery was 71 min. All patients were treated using straight, nonroticulating laparoscopic instruments. Mean tumor diameter was 6 cm (range: 5–12 cm). In total, 5 patients underwent cystectomy, 3 unilateral salpingo-oopherectomies (USO), 1 bilateral salpingo-oopherectomy (BSO), 1 USO + intraligamentary myomectomy, and 2 salpingectomies. In 2 of the patients, cholecystectomy (USO + cholecystectomy) and appendectomy (cystectomy + appendectomy) were performed concomitantly. All patient pathology reports were benign. None of the patients converted to laparotomy. All patients were discharged on postoperative d1. None of the patients required readmission to hospital. After surgery all patients reported that they were satisfied with their incision and cosmetic results, and none of the patients experienced any wound problem (Figures 4 and 5).

## 4. Discussion

SILS is a promising form of minimally invasive surgery and is currently in the initial stages of clinical use. There is growing interest in and enthusiasm for SILS among surgeons, patients, and the medical industry [1, 2]. The first single-port appendectomy was performed in 2005, followed by the first single-port cholecystectomy in 2007. Today, complex urological, gynecological, colorectal, and bariatric surgical procedures have been performed using the SILS technique and equipment. Use of SILS has been facilitated by the introduction of rotating and curved instruments into clinical practice [11–14]. On the other hand, new surgical devices, including expensive single ports, roticulating devices, and curved instruments, may limit the widespread use of SILS. If the technical difficulties associated with SILS could be overcome using less expensive conventional laparoscopic instruments, this novel surgical approach may become more common, without extra cost or lesser cost [15].

Following the introduction of SILS, some surgeons modified the approach and produced their own single-port access devices using surgical gloves. Hayashi et al. proved the effectiveness of a self-made surgical glove port for SILS in 23 patients. They made a 1.5 cm skin incision on the umbilicus, and then a small wound retractor was installed in the umbilical wound. Next, a nonpowdered surgical glove was placed on the wound retractor through which three 5 mm slim trocars were inserted via the fingertips. Surgery in all 23 cases was successful without the occurrence of intra- or postoperative complications [16]. Moreover, other studies reported an approach using a single port in the umbilicus and triangular classical trocars [1, 2, 17].

In relative terms, there are currently only a small number of reports of adnexal masses treated via SILS using straight classical laparoscopic instruments. Herein we described a modification of SILS surgery that eliminates the necessity of using expensive roticulating devices. In the present study, we used the SILS port and conventional, straight laparoscopic instruments. SILS is associated with some limitations, such as the close proximity of the working instruments, limited triangulation of the instruments, limited range of motion, an unstable camera platform, and often a small number of ports. In fact, the term “sword fighting” was used to describe instrument collision during SILS. Such limitations make SILS difficult and are associated with prolonged surgical duration, as compared to conventional laparoscopy [15, 17]. Paek et al. used a special Alexis wound retractor and a homemade single multichannel port access system for SILS hysterectomy. They reported that collision between the camera and surgical instruments was a major problem during the procedure and suggested using a 5 mm endoscope with an angle of 30 degrees, as it provides a wider field of vision [17].

In the present study, we used a 10 mm endoscope with an angle of 0 degrees and did not encounter any serious problems, although we do acknowledge having some difficulty due to collision of the instruments and camera. The most important problem we encountered during surgery was the collision of the conventional laparoscopic device and limited space for instrument movements; however, these difficulties never resulted in an aborted or cancelled procedure. Although instrument collision was a major problem during this procedure, it was overcome by repositioning the instruments and/or the surgeon; positioning the surgeon at the patient's head rather on the lateral side was an effective solution to instrument collision, making this procedure much easier. However, to prevent any intra- and postoperative complications related to instrument collision, surgeons should carefully perform these operations.

The most important part of the usage of the straight laparoscopic instrument in SILS surgery was the easy transfer of the oldest experience with these surgical devices. In the present study, laparoscopic treatment of adnexal masses using the SILS port and standard, straight laparoscopic instruments was successful in all 14 patients. Garcia-Henriquez et al. reported that SILS cholecystectomy is feasible using standard, straight surgical instruments and that use of the SILS port decreased back end instrument collisions and facilitated better separation between the trocar heads and platform, as compared to using 3 individual ports in a single incision [17]. Akgür et al. described single-port incisionless intracorporeal conventional equipment endoscopic appendectomy (SPICES). The researchers used an 11 mm conventional port (that did not require an incision beyond the umbilicus) and conventional working instruments [6]. Supraumbilical, infraumbilical, or transumbilical incisions can be used for SILS. It is generally accepted that a transumbilical incision, rather than a supra- or infraumbilical incision, results in a more cosmetically pleasing scar and an almost normal-looking umbilicus [14]. In the present study, the transumbilical approach was used, and in all 14 patients the incision was 2.0–2.5 cm, as previously reported [14].

Tam et al. reported that SILS appendectomy using conventional instruments in children was feasible. They concluded that use of conventional instruments in SILS is technically possible in children undergoing simple to complex procedures and may have the potential to popularize this approach by eliminating the mandatory demand for specially designed instruments [5]. SILS was initially performed by crossing roticulating and articulating laparoscopic instruments. Some researchers suggested using 1 roticulating instrument and 1 straight instrument for dissection [5, 18, 19]. Use of roticulating and articulating devices is complicated due to the difficult hand-eye coordination and limited surgical space, and use of conventional straight instruments may overcome this difficulty; however, use of conventional instruments also has some drawbacks, including instrument collision, limited instrument triangulation, limited range of motion, and often a small number of ports [17].

Tam et al. reported that crossing 2 straight instruments was not significantly different than conventional laparoscopic skills and that the instruments may need to be moved between hands during surgery. In the present study, we also frequently changed the placement of surgical instruments, which we think may have helped in overcoming the problem of instrument collision [5]. Podolsky and Curcillo II reported their 2-year experience with more than 100 SILS procedures; their major technical refinement was the transition from special roticulating instruments to conventional straight instruments [20].

In the present study, we performed 1 cholecystectomy and 1 appendectomy concomitantly with ovarian cystectomy and unilateral salpingo-oopherectomy, respectively, via the same umbilical incision; the ability to perform multiple procedures via a single incision is an advantage which SILS has over the classical laparoscopic approach. Surico et al. reported concomitant ovarian cystectomy and cholecystectomy using a multi-instrument access port and concluded that single-port surgery eliminates the problem of multiple site placement of accessory ports [21]. On the other hand, Hart et al. reported concomitant SILS cholecystectomy and hysterectomy for the treatment of a symptomatic fibroid uterus and symptoms of cholelithiasis in a 37-year-old woman. They concluded that complex concomitant procedures could be performed using the SILS approach [22]. SILS reduces the number of trocars used in classical multiport laparoscopic surgery [20].

The significance and importance of any new surgical approach are dependent upon its widespread acceptance and use in a large number of patients. The cost and availability of new instruments, the need surgeon retraining, and efficacy and safety are all important factors that determine the level of acceptance of any new technique [5]. This approach may help increase the popularity of SILS for adnexal masses.

Umbilical hernia is a concern about SILS surgery due to the relatively large umbilical incision. Gunderson et al. retrospectively reviewed the 211 women who underwent SILS surgery for a benign or malignant gynecologic indication via a single 1.5 to 2.0 cm umbilical incision. After a median postoperative follow-up time of 16 months, 2.4% of the patients developed umbilical hernia. However, majority of these women (4/5) had some significant risk factors for fascial weakening independent of LESS, like requirement for a second abdominal surgery and a cancer diagnosis with postoperative chemotherapy administration. When these subjects deemed “high risk” for incisional disruption were excluded from the analysis, the umbilical hernia rate was 0.5% (1/207). The authors concluded that the overall umbilical hernia rate was 2.4% and was lower (0.5%) in subjects without significant comorbidities [23]. However, further studies with larger sampler size and longer follow-up are needed to reach clear conclusions on this debate.

Another important concern is the prolongation of the operative time in SILS surgery. Lee et al. compared perioperative outcomes of single port access laparoscopic adnexal surgery versus conventional laparoscopic adnexal surgery. In this study, there were no differences between SPA and conventional groups in median operation time (64 min versus 57.5 min, *P* = 0.252) [8]. Park et al. reported that operative time was 60 minutes (27–245), 105 minutes (50–185), and 60 minutes (30–115) for an oophorectomy, cystectomy, and salpingectomy, respectively [24]. Also, Jung et al. reported that mean duration of single port adnexal surgery was 64.5 min (range 21–176 min) similar to our experience [9]. However, it has been also reported that duration of operation decreases by the end of the learning curve and that in an experienced hands duration of operation will not increase too much [25].

Although we did not perform a comparative study, we observed that single port incision has a better cosmetic outcome compared with traditional laparoscopic surgery Also, patients satisfaction was very good in patients who underwent SILS surgery. However, further comparative studies between classical laparoscopic surgery and SILS surgery with larger sample size are needed to reach clear conclusion about the cosmetic outcome.

Review of the literature in Table 2 showed that single port management of benign adnexal masses is feasible without increasing complication rates. A relatively increased duration of operation might be related to learning curve and instrument collision. However, umbilical incision might reduce the risk of tumor spillage related to cyst rupture. However, of properly designed comparative studies with single port and classic laparoscopic surgery are urgently needed.

## 5. Conclusion

We think that this procedure described herein is feasible for the treatment of adnexal masses and is more cost effective than standard SILS; however, it is associated with some difficulties, including the collision of straight laparoscopic instruments. The present study is limited by its retrospective design and limited samples size, and further prospective studies with larger sample size are needed to reach more clear conclusions. Additional research is needed to more clearly discern the safety and benefit of this approach. Also, confirmation of SILS superiority to other minimal invasive laparoscopic approaches needs to be confirmed in prospective randomized studies. Furthermore, this approach should also be validated for other commercial ports.

*Condensation.* Removal of adnexal masses via single-incision laparoscopic surgery using a combination of the SILS port and straight nonroticulating laparoscopic instruments is feasible.

## Figures and Tables

**Table 1:** Characteristics of the patients.

Patients no	Age (years)	Menopausal status	Size and features of adnexal mass	Type of operation	Duration of operation (minutes)	Pathology
1	61	Postmenopausal	7 cm trilobulated and septated ovarian cyst	BSO	85	Serous cystadenoma
2	52	Postmenopausal	7 cm solid cystic ovarian cyst	USO	70	Serous cystadenoma
3	42	Postmenopausal	5 cm complex ovarian cysts on left ovary	USO + Adhesiolysis	60	Serous cystadenoma
4	39	Premenopausal	12 cm endometrioma	Cystectomy + Adhesiolysis	130	Endometrioma
5	34	Premenopausal	5 cm ruptured ovarian cysts with massive hemoperitoneum	Cystectomy	55	Corpus hemorhagicum
6	28	Premenopausal	5 cm complex ovarian cysts	Cystectomy	60	Endometrioma
7	21	Premenopausal	5 cm ruptured ovarian cysts with massive hemoperitoneum	Cystectomy	60	Corpus hemorhagicum
8	28	Premenopausal	5 cm ruptured ovarian endometrioma	Cystectomy	80	Endometrioma
9	33	Premenopausal	4 cm adnexal mass	Salpingectomy	50	Ectopic pregnancy
10	36	Premenopausal	6 cm Tubo-ovarian abscess	Salpingectomy	45	Tubo-ovarian abscess
11	46	Premenopausal	8 cm complex adnexal mass	USO + Intraligamentary myomectomy	50	Serous cyst + leiomyoma
12	66	Postmenopausal	7 cm complex ovarian cysts	USO	130	Mucinous cystadenoma + Cholecystitis
13	28	Premenopausal	5 cm ruptured ovarian cysts with massive hemoperitoneum	Cystectomy + appendectomy	90	Corpus hemorhagicum + appendicitis
14	24	Premenopausal	9 cm endometrioma	Cystectomy	65	Endometrioma

BSO: bilateral salpingo-oopherectomy.

USO: unilateral salpingo-oopherectomy.

**Table 2:** Review of the literature of single port laparoscopy in the management of adnexal masses.

Author	Country, year	*n*	Type of port	Size of the adnexal mass, size (range)	Duration of operation, minutes (range)	Complication	Conclusion
Kim et al. [4]	Korea, 2009	24	Homemade glove port	5 cm (3–10)	70 (40–128)	—	Feasible
Escobar et al. [3]	USA, 2010	8	Multichannel port	—	—	—	Additional investigation is needed
Lee et al. [8]	Korea, 2010	17	Homemade glove port	—	—	—	Comparable operative outcomes
Jung et al. [9]	Korea, 2011	86	Homemade glove port	6	64 (21–176)	—	Feasible
Kim et al. [10]	Korea, 2011	94	Homemade glove port	6	50	—	Safe and feasible
Current	Turkey, 2012	14	SILS Port	6 (5–12)	71 (45–130)	—	Feasible
